# Translational and post-translational regulation of mouse cation transport regulator homolog 1

**DOI:** 10.1038/srep28016

**Published:** 2016-06-15

**Authors:** Yuki Nomura, Yoko Hirata, Kazutoshi Kiuchi, Kentaro Oh-hashi

**Affiliations:** 1 United Graduate School of Drug Discovery and Medical Information Sciences, Gifu University, 1-1 Yanagido, Gifu 501-1193, Japan; 2 Department of Chemistry and Biomolecular Science, Faculty of Engineering, Gifu University, 1-1 Yanagido, Gifu 501-1193, Japan

## Abstract

Cation transport regulator homolog 1 (Chac1) is an endoplasmic reticulum (ER) stress inducible gene that has a function as a γ-glutamyl cyclotransferase involved in the degradation of glutathione. To characterize the translation and stability of Chac1, we found that the Kozak-like sequence present in the 5′ untranslated region (5′UTR) of the Chac1 mRNA was responsible for Chac1 translation. In addition, the short form (ΔChac1), which translated from the second ATG codon, was generated in the absence of the 5′UTR. The proteasome pathway predominantly participated in the stability of the Chac1 protein; however, its expression was remarkably up-regulated by co-transfection with ubiquitin genes. Using an immunoprecipitation assay, we revealed that ubiquitin molecule was directly conjugated to Chac1, and that mutated Chac1 with all lysine residues replaced by arginine was also ubiquitinated. Finally, we showed that WT Chac1 but not ΔChac1 reduced the intracellular level of glutathione. Taken together, our results suggest that the Chac1 protein expression is regulated in translational and post-translational fashion due to the Kozak-like sequence in the 5′UTR and the ubiquitin-mediated pathways. The bidirectional roles of ubiquitination in regulating Chac1 stabilization might give us a new insight into understanding the homeostasis of glutathione under pathophysiological conditions.

The ubiquitin-proteasome pathway is a proteolytic mechanism that specifically ubiquitinates protein substrates targeted for degradation by the proteasome^1^. This pathway is known to associate with endocytosis of membrane proteins, specific protein-protein interactions for signal transduction, DNA repair and gene transcription^2^,^3^,^4^. Protein ubiquitination forms an isopeptide bond between the C-terminal carboxyl group of ubiquitin and the ε-amino group of lysine residues on target proteins. The successive linkage of ubiquitin to a protein substrate with free ubiquitins through its lysine residues forms a polyubiquitin chain. In the course of ubiquitin conjugation, a ubiquitin activating enzyme (E1) phosphorylates the carboxylic group of ubiquitin and binds the phospho-ubiquitin to the active cysteine site. This is followed by the transfer of the activated ubiquitin to the cysteine residue of ubiquitin conjugating enzyme (E2) through a trans(thio)esterification reaction. Through the assistance of ubiquitin ligase (E3), the ubiquitin molecule is transferred to a lysine residue on the protein substrate. It is presumed that more than 600 types of E3 ubiquitin ligases exist in the human genome and that each one of them recognizes specific substrates, suggesting that different types of ubiquitin-conjugation reactions play key roles in regulating a variety of cellular events, including signal transduction, gene transcription and the cell cycle^5^.

A ubiquitin molecule contains seven lysine residues (K6, K11, K27, K29, K33, K48 and K63); thus, it is considered that there are seven different types of isopeptide linkages. Polyubiquitin chains are usually composed of only one type of isopeptide linkage; however, more than two types of isopeptide linkages (i.e., heterogeneous chains) have been identified^6^,^7^,^8^. It is reported that the K48-linked polyubiquitin chain is recognized by the proteasome, leading to the degradation of the substrate protein^6^,^9^. On the other hand, the K63-linked polyubiquitin chain participates in endosomal trafficking, signal transduction, and DNA repair but not in protein degradation^2^,^3^,^10^. In addition, the heterogeneous chains formed by certain pairs of E2 and E3 are reported to synthesize non-degradable proteins^8^. Moreover, certain proteasome degradation processes are independent of lysine residues on the substrate protein (i.e., non-canonical ubiquitination)^11^. For instance, the degradation of p21, ERK3 and Cyclin G1 by the proteasome is mediated by the ubiquitination at the N-terminal methionine instead of lysine residues^12^,^13^,^14^,^15^. Furthermore, the hydroxyl groups of serine and threonine residues and the thiol group of cysteine residues are found to be potential sites of ubiquitination^16^,^17^,^18^,^19^.

Chac1 was first identified as a novel ER stress inducible gene in human aortic endothelial cells treated with oxidized phospholipids^20^,^21^. Until now, it has been reported that various stimuli triggering ER stress up-regulated Chac1 mRNA^21^,^22^,^23^,^24^,^25^,^26^,^27^,^28^. Activating transcription factor 4 (ATF4) plays a key role in regulating the expression of Chac1 by directly binding to the ATF4-consensus sequence in the 5′ flanking region of the Chac1 gene^28^,^29^,^30^. ATF4 is among the three canonical ER resident stress sensors, PERK-ATF4, IRE1-sXBP1 and ATF6. Moreover, the expression level of Chac1 mRNA is increased in breast and ovarian cancers associated with poor prognosis, and knockdown of Chac1 results in decreased cell migration^31^. Chac1 mRNA is also up-regulated in *Shigella*-infected HeLa cells and duck hepatitis A virus genotype C-infected duck liver^32^,^33^. In regard to functional capability, it has been reported that Chac1 functions as a γ-glutamyl cyclotransferase that specifically degrades glutathione by its catalytically active residue at E116 in the mouse and E115 in the human protein^30^,^34^,^35^. Furthermore, Chac1 plays an important role in regulating neurogenesis by Notch deglycination at the E1669 residue^36^,^37^. Until now, studies regarding the transcriptional regulation and functional characterization of Chac1 have been actively performed; however, its translational and post-translational regulatory mechanisms are still poorly elucidated. We previously reported that Chac1 protein was stabilized by the treatment of a proteasome inhibitor^28^. We therefore investigated expression mechanisms of the mouse Chac1 protein by focusing on its 5′UTR region as well as ubiquitination and proteasome-mediated degradation.

## Results

### The expression level of the Chac1 protein is regulated by the proteasome pathway

**Figure 1:**
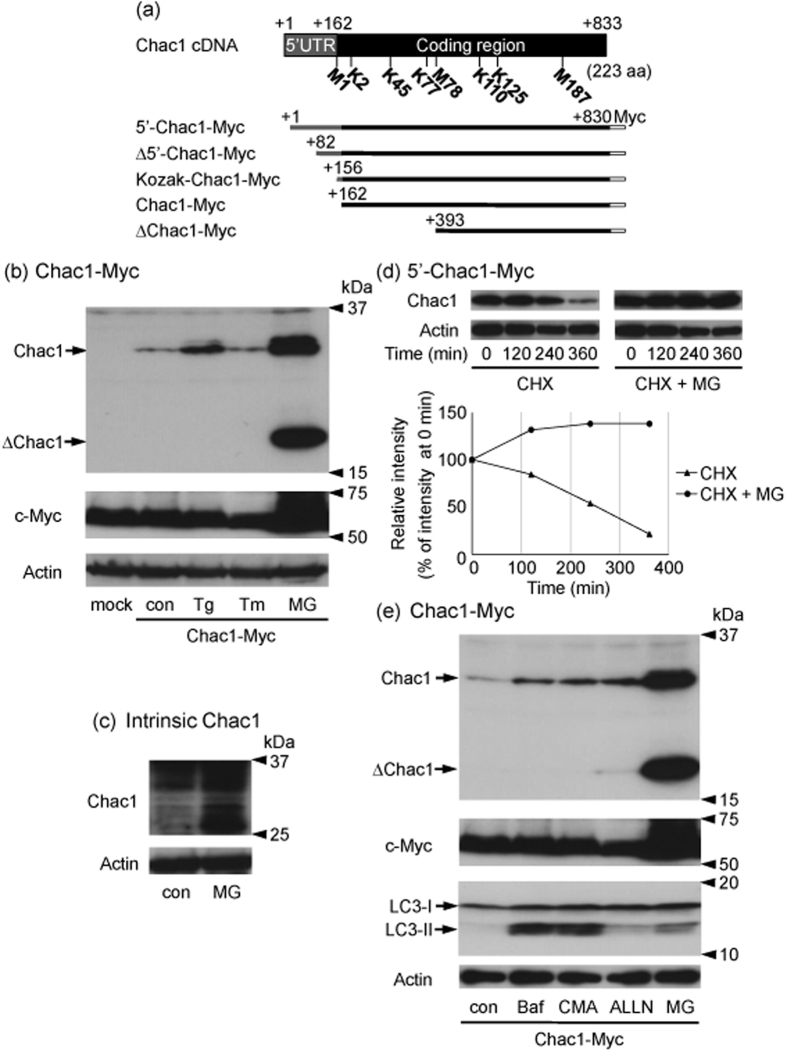
The Chac1 protein is regulated by the proteasome pathway. (**a**) Schematic representation of the Chac1 constructs showing the positions of methionine and lysine used in this study (NCBI accession No. NM_026929). (**b**) HEK293 cells were transfected with pcDNA3.1 (mock) or Chac1-Myc. After 24 h, cells were treated with vehicle (con), 0.1 μM thapsigargin (Tg), 2 μg/mL tunicamycin (Tm) or 20 μM MG132 (MG) for 12 h, and the resultant cell lysates were analyzed by western blot using c-Myc or Actin antibodies according to Materials and Methods. (**c**) HEK293 cells were treated with vehicle (con) or 20 μM MG for 12 h, and the resultant cell lysates were analyzed by western blot using Chac1 or Actin antibodies. (**d**) HEK293 cells were transfected with 5′-Chac1-Myc. After 24 h, cells were treated with 10 μg/mL cycloheximide (CHX) in the presence or absence of MG for the indicated times, and the resultant cell lysates were analyzed by western blot using c-Myc or Actin antibodies. Band intensities were analyzed by ImageJ software (National Institutes of Health, U.S.A.). (**e**) HEK293 cells were transfected with Chac1-Myc. After 24 h, the cells were treated with vehicle (con), 50 nM Bafilomycin A1 (Baf), 50 nM Concanamycin A (CMA), 10 μM *N*-Acetyl-L-leucyl-L-leucyl-L-norleucinal (ALLN) or 20 μM MG for 12 h, and the resultant cell lysates were analyzed by western blot using c-Myc, LC3 or Actin antibodies. All experimental data were obtained from three independent cultures.

The mouse Chac1 protein consists of 223 amino acids (aa). The Chac1 mRNA is coded from position 162 to 833 (+162/+833) following the 5′UTR (+1/+161) as found in the NCBI database under accession NM_026929 (Fig. 1a). We previously reported that the Chac1 protein is stabilized by the proteasome inhibitor, MG132, in Neuro2a cells transiently transfected with a Chac1 construct (+162/+833)^28^. We next focused on the 5′UTR region of the Chac1 mRNA, especially the Kozak-like sequence (GGCACC) just before the translation start site (Fig. 1a). First, we constructed Chac1 expression vectors having the entire 5′UTR (+1/+830; 5′-Chac1-Myc), a truncated 5′UTR (+82/+830; Δ5′-Chac1-Myc), the Kozak-like sequence just before the translation start site (+156/+830; Kozak-Chac1-Myc), and a vector lacking the 5′UTR (+162/+830; Chac1-Myc) (Fig. 1a and Supplementary Fig. S1). To investigate the expression of the Chac1 protein, a Chac1 vector having a Myc-epitope at the C-terminus (Chac1-Myc) was transiently transfected into HEK293 cells. The cells were subsequently treated with the ER stress inducers (thapsigargin (Tg) and tunicamycin (Tm)) or MG132 (Fig. 1b). Consistent with our previous report, the Chac1 protein was stabilized by MG132, but not by the other agents. To confirm whether intrinsic Chac1 protein is stabilized by MG132, we investigated its expression in HEK293 cells in the presence or absence of MG132 using a Chac1 antibody (Fig. 1c). As a result, the expression of intrinsic Chac1 was actually increased by treatment with MG132. We further investigated Chac1 expression in the presence or absence of cycloheximide (CHX), an inhibitor of protein translation, to determine whether the increased Chac1 expression by MG132 was due to the inhibition of proteasomal degradation. Chac1 protein was degraded in the presence of CHX in a time dependent manner, whereas it was markedly stabilized by the treatment of a combination of CHX and MG132 (Fig. 1d). As MG132 was reported to activate autophagy^38^, we examined whether autophagy takes part in regulating the expression level of Chac1 protein. HEK293 cells expressing Chac1-Myc were treated with MG132, lysosomal acidification inhibitors (Bafilomycin A1 (Baf) or Concanamycin A (CMA)), or the calpain I inhibitor *N*-Acetyl-L-leucyl-L-leucyl-L-norleucinal (ALLN). As shown in Fig. 1e, Chac1 was stabilized by MG132 treatment, whereas treatments with Baf, CMA or ALLN showed small effects. Under the same conditions, the autophagic marker LC3 conjugated to PE (PE-conjugated LC3-II)^39^, was dramatically up-regulated by treatment with Baf and CMA, whereas the effect of MG132 was to a lesser extent (Fig. 1e). In parallel, the expression of the intrinsic c-Myc protein, which is a well-known substrate of proteasomal degradation^40^,^41^, was also stabilized only by treatment with MG132. As a result, the c-Myc protein was stabilized by MG132 but not by the other agents (Fig. 1e). These data indicate that the Chac1 protein is predominantly regulated by the proteasome pathway in a post-translational manner.

### Chac1 translation is enhanced by the Kozak-like sequence in the 5′ untranslated region and the short form of Chac1 is translated from the second methionine codon

**Figure 2:**
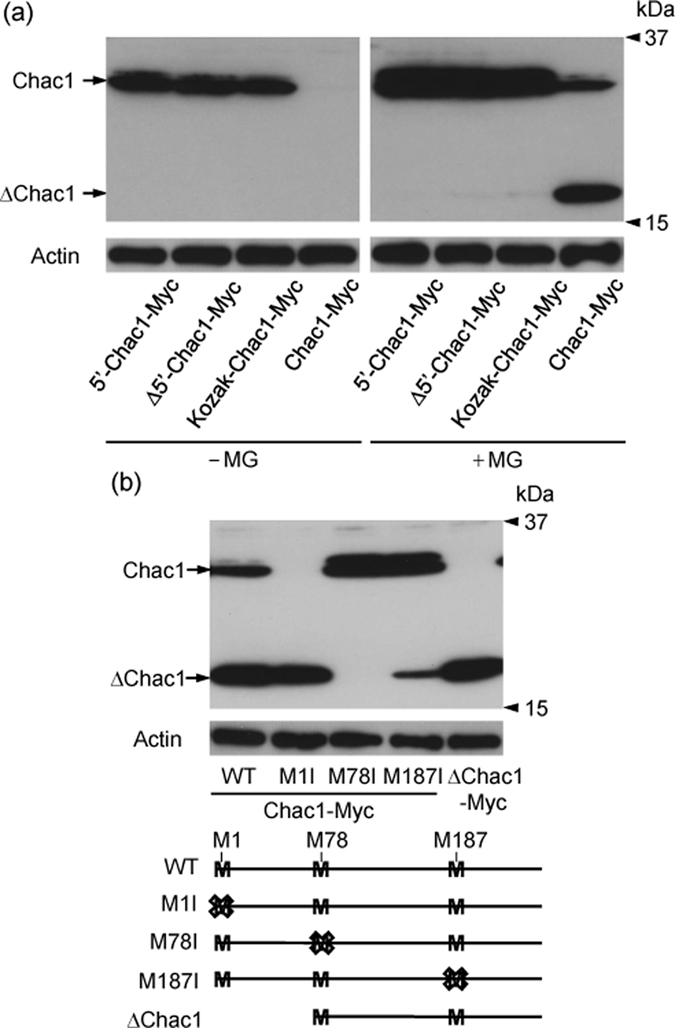
Chac1 translation is enhanced by the Kozak-like sequence in the 5′ untranslated region and the short form of Chac1 is translated from the second methionine codon. (**a**) HEK293 cells were transfected with 5′-Chac1-Myc, Δ5′-Chac1-Myc, Kozak-Chac1-Myc or Chac1-Myc. After 24 h, the cells were treated with vehicle (−MG) or 20 μM MG for 12 h, and the resultant cell lysates were analyzed by western blot using c-Myc or Actin antibodies. (**b**) HEK293 cells were transfected with Chac1-Myc (WT, M1I, M78I or M187I) or ΔChac1-Myc. After 24 h, the cells were treated with 20 μM MG for 12 h, and the resultant cell lysates were analyzed by western blot using c-Myc or Actin antibodies. All experimental data were obtained from three independent cultures.

To investigate whether the 5′UTR of Chac1 is responsible for the translation and stabilization of the protein, we transfected HEK293 cells with each of the Chac1 constructs shown in Fig. 1a. As a result of transfection, the expression level of Chac1 was markedly up-regulated in the presence of the Kozak-like sequence before the translation start site (Fig. 2a). The Chac1 protein was usually detected at approximately 30 kDa by western blot; however, another short form of Chac1 (ΔChac1) was detected at approximately 17 kDa when HEK293 cells were treated with MG132 (Figs 1b,e and  2a). As Chac1 mRNA has two additional methionine codons in its coding region (Fig. 1a), we speculated that ΔChac1 could be translated from the downstream methionine codons of a prospective translation start site. Thus, we made four mutant Chac1 constructs by replacing each methionine with isoleucine (Chac1 (M1I, M78I and M187I)-Myc) and deleting the upstream region from the second ATG codon (+393/+830; ΔChac1-Myc) (Figs 1a and 2b). We then evaluated the expression level of each construct in the presence of MG132. As shown in Fig. 2bΔChac1 was detected in HEK293 cells overexpressing Chac1 (WT)-Myc, Chac1 (M1I)-Myc, Chac1 (M187I)-Myc and ΔChac1 (+393/+830)-Myc but not in cells transfected with Chac1 (M78I)-Myc (Fig. 2b). This suggested that ΔChac1 might be translated from the second ATG codon of the Chac1 mRNA.

### Ubiquitin overexpression stabilizes Chac1 protein in HEK293 cells

**Figure 3:**
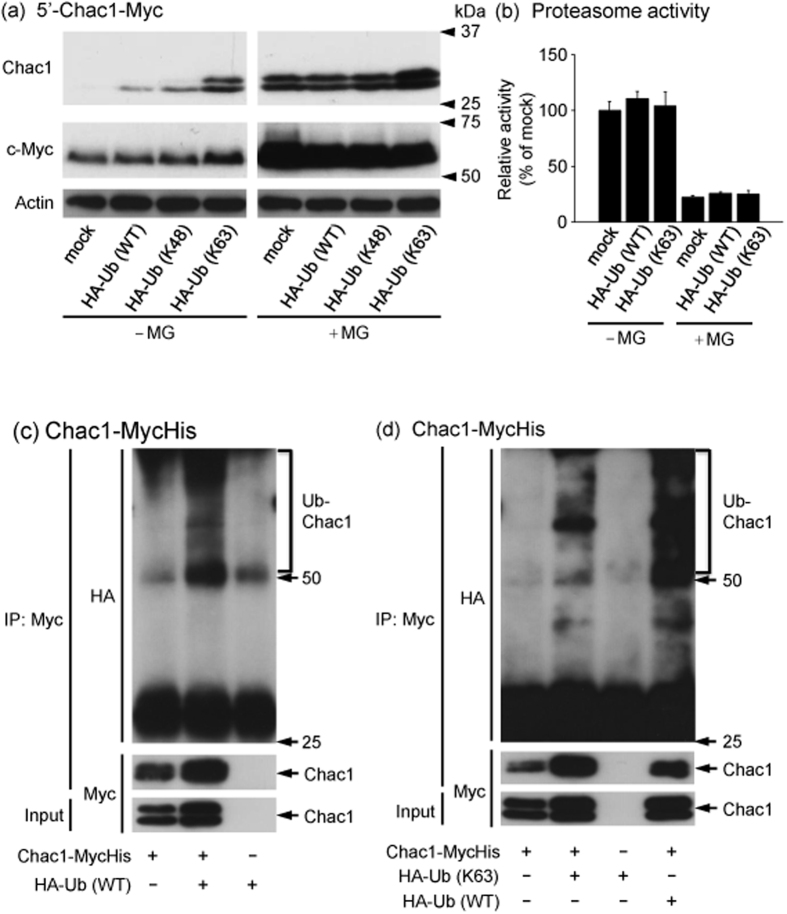
Chac1 is stabilized by ubiquitin co-expression and actually ubiquitinated in HEK293 cells. (**a**) HEK293 cells were transiently co-transfected with 5′-Chac1-Myc and HA-Ub (WT, K48 or K63). After 24 h, the cells were treated with vehicle (−MG) or 20 μM MG for 12 h, and the resultant cell lysates were analyzed by western blot using c-Myc or Actin antibodies. (**b**) HEK293 cells transfected with pcDNA3.1 (mock), HA-Ub (WT or K63). After 36 h, the cells were collected and the proteasome activity was measured according to the Materials and Methods. (**c**) HEK293 cells were transiently co-transfected with Chac1-MycHis and HA-Ub (WT). After 24 h, the cells were treated with 20 μM MG for 12 h. Myc-tagged proteins were immunoprecipitated from the resultant cell lysates using a c-Myc antibody and Protein G Sepharose and were analyzed by western blot using HA or c-Myc antibodies. Total cell lysate (Input) was analyzed by western blot using a c-Myc antibody. (**d**) HEK293 cells were transiently co-transfected with Chac1-MycHis and HA-Ub (WT or K63). After 24 h, the cells were treated with 20 μM MG for 12 h. Myc-tagged proteins were immunoprecipitated from the resultant cell lysates using a c-Myc antibody and Protein G Sepharose and were analyzed by western blot using HA or c-Myc antibodies. The total cell lysate (Input) was analyzed by western blot using a c-Myc antibody. All experimental data were obtained from three independent cultures. Ub-Chac1 indicates the ubiquitinated Chac1.

Because Chac1 was degraded by the proteasome pathway, we investigated whether the degradation of the Chac1 protein is facilitated by ectopically expressed ubiquitin. 5′-Chac1-Myc was transfected together with N-terminal HA-tagged wild type ubiquitin (HA-Ub (WT)) or single-lysine mutant ubiquitin with K48 (HA-Ub (K48)) or K63 (HA-Ub (K63)) as the only lysine residue in the ubiquitin molecule^42^. Contrary to our expectations, Chac1 was stabilized by the co-transfection of each HA-Ub construct, and the effect of HA-Ub (K63)-overexpression was prominent (Fig. 3a). The increase in Chac1-Myc expression by HA-Ub (WT) co-expression was almost at the same level as observed by HA-Ub (K48). On the other hand, the expression of intrinsic c-Myc was not elevated by the transfection of each type of HA-Ub construct (Fig. 3a). We then investigated the proteasome activity when HA-Ub (WT or K63) was transfected into HEK293 cells (Fig. 3b). As a result, the activity was not altered by the HA-Ub overexpression, whereas it was decreased by the MG132 treatment. Next, we examined whether Chac1 was directly ubiquitinated in the HA-Ub (WT) or HA-Ub (K63)-overexpressing cells using an immunoprecipitation assay. As shown in Fig. 3c,d, immunoprecipitates from cells expressing both Chac1-MycHis and HA-Ub (WT or K63) in the presence of MG132 showed a ladder of higher molecular size bands detected by an anti-HA antibody. Interestingly, the band derived from the cells expressing HA-Ub (K63) was weaker compared to HA-Ub (WT) (Fig. 3d).

### Mutant Chac1 is still ubiquitinated in HEK293 cells and stabilized in a similar fashion as wild type Chac1 after substitution of all lysine residues for arginine

**Figure 4:**
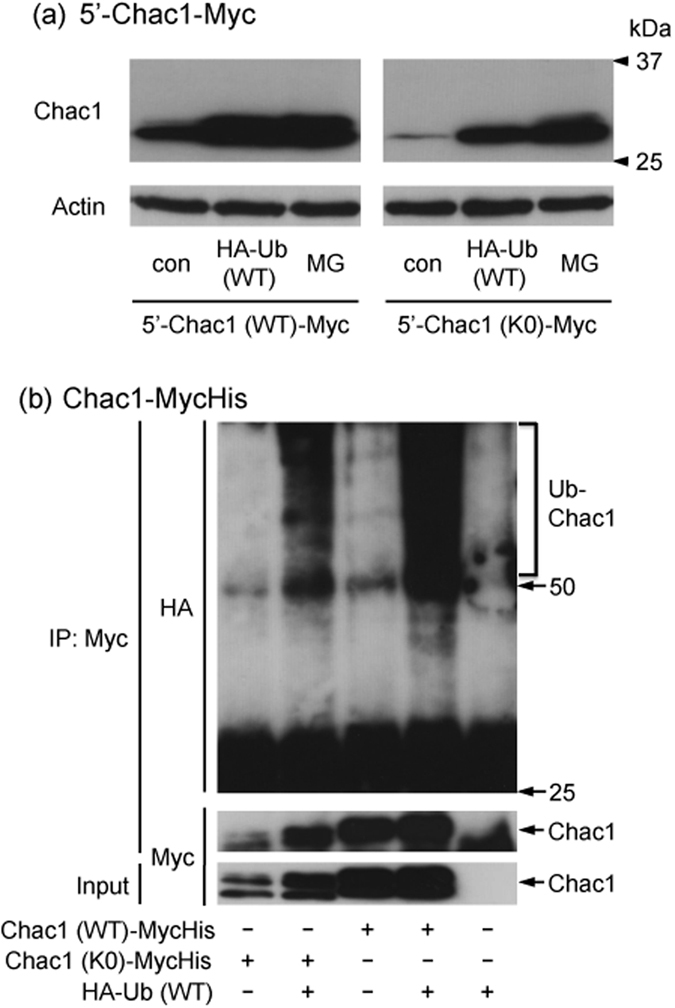
Chac1 (K0) devoid of lysines is still ubiquitinated in HEK293 cells. (**a**) HEK293 cells were transiently co-transfected with 5′-Chac1 (WT or K0)-Myc and HA-Ub (WT). After 24 h, the cells were treated with vehicle (con) or 20 μM MG for 12 h, and the resultant cell lysates were analyzed by western blot using c-Myc or Actin antibodies. (**b**) HEK293 cells were transiently co-transfected with Chac1 (WT or K0)-MycHis and HA-Ub (WT). After 24 h, the cells were treated with 20 μM MG for 12 h. Myc-tagged proteins were immunoprecipitated from the resultant cell lysates using a c-Myc antibody and Protein G Sepharose and were analyzed by western blot using HA or c-Myc antibodies. The total cell lysate (Input) was analyzed by western blot using c-Myc antibody. Figure 4a,b data were obtained from three and two independent cultures, respectively. Ub-Chac1 indicates the ubiquitinated Chac1.

As Chac1 was directly ubiquitinated in HA-Ub overexpressing cells, we next investigated whether the ubiquitin molecule conjugated to the lysine residues in Chac1 as observed in many other proteins. To address this issue, we made a construct of mutated Chac1 (+1/+830; 5′-Chac1 (K0)-Myc) where all lysine residues were replaced with arginines because the mouse Chac1 protein has five lysine residues in the coding region (Fig. 1a). HEK293 cells were then transfected with 5′-Chac1 (WT)-Myc or 5′-Chac1 (K0)-Myc together with HA-Ub (WT) and incubated in the presence or absence of MG132 (Fig. 4a). The expression level of 5′-Chac1 (K0)-Myc as well as 5′-Chac1 (WT)-Myc was increased by co-transfection of HA-Ub (WT) and by MG132 treatment. Therefore, we examined whether the mutant Chac1 (K0) was ubiquitinated or not. As shown in Fig. 4b, the HA-positive molecules were detected in the Myc-immunoprecipitate derived from the cells co-expressing Chac1 (K0)-MycHis and HA-Ub (WT) in the presence of MG132.

### The N-terminal region of Chac1 is not associated with either stabilization by ubiquitin co-expression or degradation by the proteasome pathway

**Figure 5:**
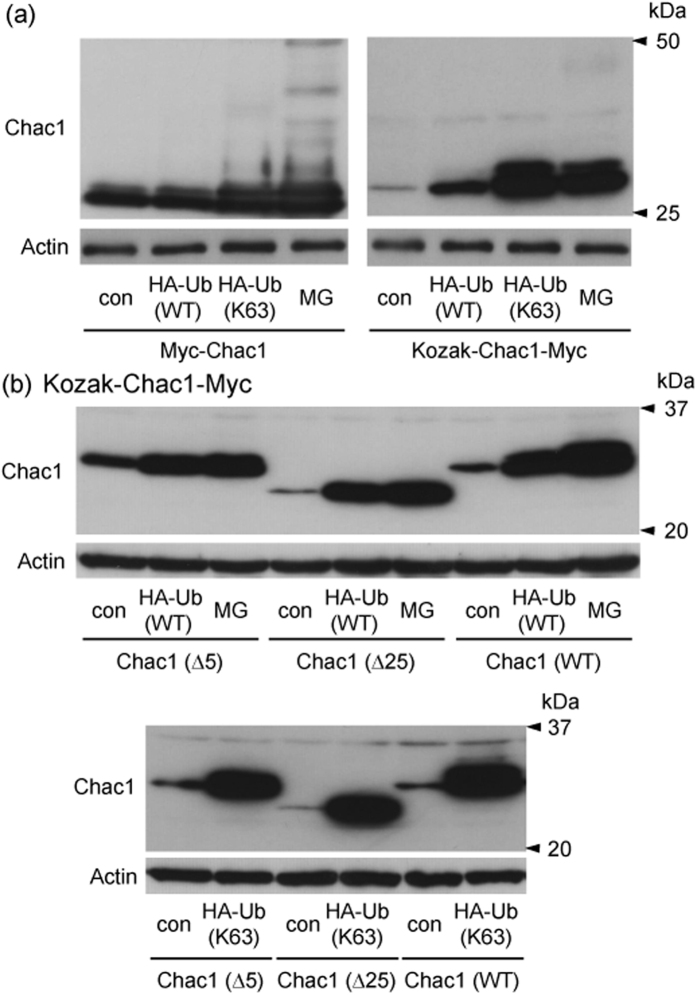
The N-terminal region of Chac1 is not associated with either stabilization by ubiquitin co-expression or degradation by the proteasome pathway. (**a**) HEK293 cells were transiently co-transfected with Myc-Chac1 or Kozak-Chac1 (WT)-Myc in addition to HA-Ub (WT or K63). After 24 h, the cells were treated with vehicle (con) or 20 μM MG for 12 h, and the resultant cell lysates were analyzed by western blot using c-Myc or Actin antibodies. (**b**) HEK293 cells were transiently co-transfected with Kozak-Chac1 (Δ5, Δ25 or WT)-Myc in addition to HA-Ub (WT or K63). After 24 h, the cells were treated with vehicle (con) or 20 μM MG for 12 h, and the resultant cell lysates were analyzed by western blot using c-Myc or Actin antibodies. All experimental data were obtained from three independent cultures.

It has been reported that the degradation of p21, ERK3 and Cyclin G1 through the proteasome pathway is triggered by ubiquitination at the N-terminal methionine instead of lysine residues^12^,^13^,^14^,^15^. As these proteins were stabilized by the addition of the Myc-epitope to the N-terminal methionine, we constructed N-terminal Myc-tagged Chac1 (+162/+833; Myc-Chac1) with the Kozak sequence (CCCACC) just before the Myc-epitope sequence. We then examined whether the expression levels of Myc-Chac1 and Kozak-Chac1-Myc were affected by co-transfection of HA-Ub (WT or K63) or by MG132 treatment. As shown in Fig. 5a, the expression level of Myc-Chac1 was much higher than that of Kozak-Chac1-Myc in the absence of MG132, and the co-transfection of HA-Ub (K63) and the MG132 treatment also increased each of the expression levels, respectively. A ladder of higher molecular weight bands were observed in both cases of HA-Ub (K63) co-transfection and MG132 treatment in the Myc-Chac1-expressing cells. The Chac1 protein has a lysine residue at the position next to the first methionine (2 aa) and its N-terminal region contains a proline-rich region (10–24 aa), which is well conserved between the mouse and human genes. Therefore, we investigated whether the N-terminal region of Chac1 contributes to its stabilization. We constructed two types of N-terminal deletion constructs: Kozak-Chac1 (Δ5)-Myc and Kozak-Chac1 (Δ25)-Myc, lacking 2–6 aa and 2–26 aa, respectively. As shown in Fig. 5b, the expression of each truncated protein was similarly influenced by Ub-overexpression and MG132 treatment compared with WT Chac1.

### Overexpression of Chac1 but not ΔChac1 significantly decreases the intracellular level of glutathione

**Figure 6:**
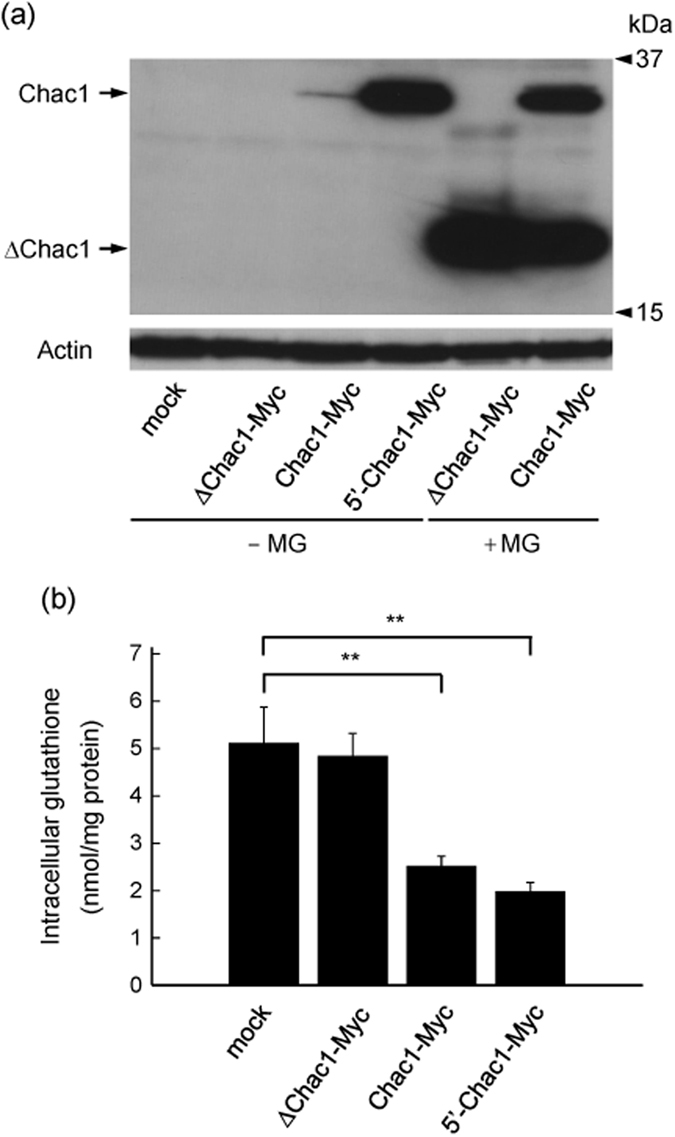
Chac1 overexpression significantly decreases the level of intracellular glutathione whereas ΔChac1 fails to change it. (**a**) HEK293 cells transfected with pcDNA3.1 (mock), ΔChac1-Myc, Chac1-Myc or 5′-Chac1-Myc. After 24 h, the cells were treated with vehicle (-MG) or 20 μM MG for 12 h, and the resultant cell lysates were analyzed by western blot using c-Myc or Actin antibodies. Experimental data were obtained from three independent cultures. (**b**) HEK293 cells were transfected with pcDNA3.1 (mock), ΔChac1-Myc, Chac1-Myc or 5′-Chac1-Myc. After 36 h, cells were collected, and the level of intracellular glutathione was measured. Values represent the mean ± SD obtained from three independent cultures. **p < 0.01 using one way-ANOVA followed by Tukey’s test.

It has been reported that Chac1 specifically degrades intracellular glutathione through its catalytic active site at E116 in mice or E115 in humans^30^,^34^,^35^. Finally, to determine whether ΔChac1 translated from the second methionine codon has catalytic activity, we transfected ΔChac1-Myc, Chac1-Myc and 5′-Chac1-Myc into HEK293 cells. As shown in Fig. 6a, the expression level of each type of Chac1 protein was found to be quite different. Chac1-Myc and 5′-Chac1-Myc significantly decreased the intracellular level of glutathione to a similar extent (^**^p < 0.01), whereas ΔChac1-Myc did not degrade glutathione even though it contains a catalytic active site (E116) (Fig. 6b).

## Discussion

In this study, we show that the Kozak-like sequence in the 5′UTR region of the Chac1 mRNA increases the expression level of the Chac1 protein and is responsible for the translation of the protein from the first methionine codon. In the absence of this sequence, the second ATG codon of the Chac1 mRNA can become a translation start site to produce the short form (ΔChac1). Our results also indicate that the expression level of the Chac1 protein is predominantly regulated by the proteasome pathway. Though it is well known that protein degradation by the proteasome pathway is followed by the sequential conjugation of ubiquitin molecules catalyzed by specific ubiquitin ligases, the co-transfection of the ubiquitin constructs WT, K48 or K63 increased the expression level of the Chac1 protein (Fig. 3). Surprisingly, the expression levels of the mutant Chac1 protein of K0 as well as those of Δ5 and Δ25 are up-regulated by ubiquitin co-expression (Figs 4 and 5). We also reveal that the intracellular level of glutathione is significantly decreased by the overexpression of full-length Chac1 but not ΔChac1 containing the active catalytic site E116.

We previously reported that the Chac1 protein is stabilized by MG132 treatment in Neuro2a cells transiently transfected with the Chac1 construct (+162/+833)^28^. Consistent with our previous report, the expression level of the Chac1-Myc protein was elevated by MG132 in overexpressing HEK293 cells and the intrinsic Chac1 protein was also increased in the presence of MG132 (Fig. 1b,c). Thus, endogenous Chac1 as well as exogenous one was similarly regulated by the proteasome pathway. In contrast to the MG132-mediated stabilization of Chac1 in a post-translational manner (Fig. 1d), only a small portion of Chac1 was found to be degraded by autophagy because treatment with Baf and CMA, which are known to attenuate autophagic degradation through the inhibition of lysosomal acidification, slightly increased the expression level of Chac1 (Fig. 1e). Treatment with ALLN under the present experimental conditions may reflect its influence on proteasomal activity as ALLN is commonly used as a calpain I inhibitor and is reported to suppress trypsin-like, chymotrypsin-like and caspase-like peptidases in the proteasomes^43^,^44^,^45^.

In this study, we detected two proteins around 30 kDa as Chac1. We therefore investigated an effect of a *N*-glycosylation inhibitor, Tm, on the expression of Chac1 protein, but the band shift was not observed (Fig. 1b). Additionally, each type of Chac1 having a Myc-epitope at the N- or C-terminus showed two bands by western blot (Fig. 5a), suggesting that the Chac1 protein could not be cleaved around the either terminal. Considering the molecular size of ubiquitin, neither of bands seems to be ubiquitin-adducts. It is reported that various kinds of proteins are subject to a variety of post-translational modifications (e.g., phosphorylation, acetylation and so on) to modulate their functions. Therefore, we intend to characterize the two bands in more detail to understand the enzymatic regulation of Chac1 in our future study.

It has been reported that the Kozak sequence adjacent to the ATG codon facilitates translation^46^. Comparing the nucleotide sequences of Chac1 5′UTR among mouse, rat and human, the Kozak-like sequence and N-terminal region of these Chac1 genes are well conserved (Supplementary Fig. S1), and we revealed that the Kozak-like sequence plays an important role in facilitating the expression of Chac1 protein. With a view to revealing whether Chac1 is translationally regulated by the Kozak-like sequence, we compared the amount of Chac1 mRNA derived from each of the transfected genes (Supplementary Fig. S2). As a result, the Chac1 mRNAs from Kozak-Chac1-Myc or Chac1-Myc were expressed to the same extent, however, the expression levels of 5′-Chac1-Myc or Δ5′-Chac1-Myc were lower than those of Kozak-Chac1-Myc or Chac1-Myc, respectively. These results indicated that other regions of Chac1 5′UTR in addition to its Kozak-like sequence might contribute to the Chac1 expression. We here demonstrated that the Kozak-like sequence has a crucial role in enhancing the Chac1 translation, but the regulation of Chac1 expression by its 5′UTR seems complicated.

Despite the increased expression of Chac1 by MG132, Chac1 was also up-regulated by ubiquitin co-expression, and it actually became ubiquitinated in HEK293 cells (Fig. 3). On the other hand, we observed that the ubiquitin-overexpression did not influence the proteasome activity. It is therefore unclear how Chac1 ubiquitination determines the fate of degradation or stabilization. In contrast to the transfected Chac1, the amount of intrinsic c-Myc remained almost unchanged by ubiquitin co-expression even though it was up-regulated by MG132 (Fig. 3a). Considering the higher transfection efficiency of HEK293 cells, we exclude the possibility that the overexpressed ubiquitin is not enough to increase the intrinsic c-Myc expression. These results imply that the stabilizations of Chac1 and c-Myc are regulated in a different manner even though both are proteasomal substrates. In addition, the overexpression of HA-Ub (K63) showed a prominent effect on Chac1 expression; however, the K63-ubiquitination of Chac1 detected by immunoprecipitation was weaker than the WT-ubiquitination. Chac1 stabilization by the HA-Ub (K63) co-expression might involve an unknown mechanism that remains to be determined. In some cases, such as the NF-κB and Nrf2 pathways, it has been demonstrated that proteasomal degradation of regulatory factors followed by their ubiquitination play a key role in the stabilization of the associated functional molecules^47^,^48^. On the other hand, it has been reported that the degradation and stabilization of the same protein [proteasome substrates such as Myc by SCF (Fbw7) and SCF (β-TrCP) and Cryptochromes by FBXL3 and FBXL21, respectively] are regulated by different ubiquitin ligase complexes through different linkage types of polyubiquitination^49^,^50^. These phenomena may explain why Chac1 was stabilized by ubiquitin co-expression despite its predominant degradation in the proteasome. Therefore, identification of the Chac1-specific ubiquitin ligases is necessary to uncover its post-translational regulation. Moreover, it has been reported that E3-E2 pairs, such as the U-box E3 ubiquitin ligase CHIP with the E2 ubiquitin conjugating enzyme UbcH5, form different types of polyubiquitin chains that contain all seven possible ubiquitin-isopeptide linkages and that the heterogeneous linkage inhibits the substrate protein degradation by the proteasome^8^. Therefore, further studies using different ubiquitin constructs may give an insight into understanding which type of polyubiquitin chain conjugates to stabilize or degrade the Chac1 protein.

Here, we demonstrated that the Chac1 mutant, where all lysine residues were replaced with arginine, met a similar fate as WT Chac1 under the same conditions of proteasome inhibition and Ub-overexpression. We furthermore constructed a Chac1 (K0) gene having a Myc-epitope in which a lysine residue was substituted with an arginine one (Chac1 (K0)-Myc(KR)His) though a Myc-epitope is often used to characterize protein ubiquitination and degradation as well as this study. This additional experiment showed that the expression of this lysineless Chac1 (K0)-Myc(KR)His is up-regulated by ubiquitin-overexpression and actually ubiquitinated in the presence of MG132 (Supplementary Fig. S3). These results support this finding that Chac1 ubiquitination might be actually lysine-independent. It was previously reported that the lysine substituted mutants of p21, ERK3 and Cyclin G1 were still ubiquitinated and degraded through the proteasome pathway in a similar fashion^12^,^13^,^14^,^15^. Collectively, Chac1 may be regulated by the non-canonical ubiquitination pathway. It has been reported that the N-terminal methionine of some substrate proteins is specifically ubiquitinated and that the addition of a Myc-epitope to the N-terminal methionine stabilizes the modified ubiquitin substrates^11^,^12^,^13^,^14^,^15^. In this study, the N-terminal Myc-tagged Chac1 (Myc-Chac1) and Kozak-Chac1-Myc were stabilized by the HA-Ub (K63) co-transfection and MG132 treatment, whereas the HA-Ub (WT) co-transfection hardly affected the expression level of Myc-Chac1 (Fig. 5a). On the other hand, Chac1 lacking the N-terminal region (Δ5 and Δ25) still responded to the overexpression of both HA-Ub (WT) and HA-Ub (K63), as well as MG132 treatment (Fig. 5b). These data suggest that the N-terminal region of Chac1 is not strongly associated with both stabilization through ubiquitination and degradation through the proteasome pathway even though Myc-Chac1 showed a slightly different result in this study. We also examined the property of Kozak-Chac1 without any tags, which was quite similar to that of C-terminal tagged Chac1 (Kozak-Chac1-Myc) (data not shown).

In the current study, the specific signals corresponding to ubiquitinated Chac1 were broadly detected around larger molecular weight by western blot, however its ubiquitinated forms remain to be determined. It has been reported that the hydroxyl groups of serine and threonine residues and even the thiol group of cysteine residues are potential sites for ubiquitination^16^,^17^,^18^,^19^. Therefore, these types of ubiquitination may participate in the stabilization and the proteasomal degradation of the Chac1 protein. We also analyzed the expression levels of serially deleted Chac1 (1–77 aa, 1–130 aa, 1–186 aa, 78–223 aa) and found that all deleted Chac1 proteins were stabilized under these experimental conditions (data not shown). Collectively, it is considered that there seems to be multiple ubiquitinated sites in the Chac1 protein molecule. Therefore, further characterization of the polyubiquitin chains and identification of the modified residues in Chac1 might give new insights into understanding the post-translational regulation of Chac1.

Under our present experimental conditions, the translation efficiency of 5′-Chac1-Myc was higher than that of Chac1-Myc; however, the difference in expression patterns as detected by western blot did not reflect the level of intracellular glutathione (Fig. 6). It has been reported that glutathione inhibits the enzymatic activity of γ-glutamylcysteine synthetase, the rate-limiting enzyme of glutathione biosynthesis; however, the reduction of glutathione releases γ-glutamylcysteine synthetase from this feedback inhibition^51^,^52^. Likewise, there might be some unknown mechanisms regulating Chac1 activity. Therefore, the expression level of Chac1 was not simply correlated with the level of intracellular glutathione as observed in this study. In contrast, ΔChac1 failed to degrade intracellular glutathione despite containing a catalytically active residue (E116). This result suggests that the N-terminal region of Chac1 (1–77 aa) is indispensable for the enzymatic activity of γ-glutamyl cyclotransferase to sustain its proper conformation and/or interaction with glutathione. Therefore, further analysis of the 3D protein structure of Chac1 is necessary to understand the function of this enzyme.

In conclusion, we have shown that the 5′UTR of Chac1 plays an important role in expressing the full-length and short form of Chac1, and the expression level of Chac1 protein is predominantly regulated by its ubiquitination. As Chac1 is known to attenuate the intracellular level of glutathione responsible for healing several pathological conditions induced by oxidative stress^53^,^54^,^55^, uncovering characteristic features of Chac1 will contribute to a novel therapeutic agent against oxidative stress-related diseases.

## Materials and Methods

### Materials

Thapsigargin (Tg), tunicamycin (Tm), cycloheximide (CHX), bafilomycin A1 (Baf), and *N*-Acetyl-L-leucyl-L-leucyl-L-norleucinal (ALLN) were obtained from Sigma-Aldrich (U.S.A.). MG132 (MG) and Suc-LLVY-MCA were purchased from Peptide Institute (Japan). Concanamycin A (CMA) was obtained from Wako (Japan). Antibodies against Chac1, c-Myc (9E10), HA, LC3 and Actin were purchased from Abcam (UK), Santa Cruz Biotechnology (U.S.A.), Clontech Laboratories (U.S.A.), Medical & Biological Laboratories (Japan) and Calbiochem (U.S.A.), respectively.

### Construction of plasmids

Mouse Chac1 cDNA (NCBI accession No. NM_026929) was amplified from a C57BL/6 mouse brain-derived cDNA library by polymerase chain reaction (PCR) and the Chac1 coding region (+162/+833) was cloned into pcDNA3.1 (pcDNA3.1-Chac1). To detect Chac1 protein by the c-Myc antibody, a Myc-epitope was directly fused to the C-terminus of Chac1 in pcDNA3.1-Chac1 to generate pcDNA3.1-Chac1-Myc by PCR (+162/+830; Chac1-Myc) using anti-sense primer 5′-TCACAGATCCTCTTCTGAGATGAGTTTTTGTTCGGTCAGTGCCAGAGGC-3′ (Fig. 1a). Four Chac1 expression constructs comprising the entire 5′ untranslated region (5′UTR) (+1/+830; 5′-Chac1-Myc), a truncated 5′UTR (+82/+830; Δ5′-Chac1-Myc), the Kozak-like sequence just before the translation start site (+156/+830; Kozak-Chac1-Myc) and a short form of mouse Chac1 translated from the second methionine (+393/+830; ΔChac1-Myc) were generated by PCR using pcDNA3.1-Chac1-Myc as the template (Fig. 1a). We further constructed three mutants (M1I, M78I and M187I; Fig. 2b) using pcDNA3.1-Chac1-Myc as the PCR template to replace methionine with isoleucine residues and mutant 5′-Chac1 (K0)-Myc (Fig. 4a) using pcDNA3.1-5′-Chac1-Myc as the PCR template to replace all lysine residues in the coding region with arginine. In addition, we generated Chac1 (WT, K0)-MycHis constructs by subtracting the stop codon and the 5′UTR from pcDNA3.1-5′-Chac1 (WT, K0)-Myc by PCR and cloning the resultant cDNAs into pcDNA3.1-MycHis. Two N-terminal deletion constructs lacking the 2–6 amino acid (aa) (Kozak-Chac1 (Δ5)-Myc) and 2–26 aa (Kozak-Chac1 (Δ25)-Myc) sequences of Chac1 were generated by PCR using pcDNA3.1-Kozak-Chac1-Myc as the template (Fig. 5b). To construct the N-terminal Myc-tagged Chac1 (Myc-Chac1; Fig. 5a), the pcDNA3.1-Chac1 coding region was subcloned into a pCMV-Myc vector using *Eco*R I to fuse the Myc-epitope to the Chac1 N-terminus, followed by subcloning into pcDNA3.1 using *Xba* I. The N-terminal HA-tagged wild type and single-lysine mutant ubiquitin constructs were generously provided by Dr. Kah-Leong LIM^42^.

### Cell culture and treatment

The human embryonic kidney cell line, HEK293, was maintained in Dulbecco’s Modified Eagle’s Medium containing 8% fetal bovine serum and 1% penicillin-streptomycin. Transfections of constructs used in this study were performed with polyethylenimine “Max” (Polysciences, U.S.A.)^56^. HEK293 cells were treated with Tg (0.1 μM), Tm (2 μg/mL), MG (20 μM), CHX (10 μg/mL), Baf (50 nM), CMA (50 nM) and ALLN (10 μM) for the indicated times.

### Western blot analysis

Cells were lysed in homogenate butter [20 mM Tris-HCl (pH 8.0) containing 137 mM NaCl, 2 mM EDTA, 10% glycerol, 1% Triton X-100, 1 mM PMSF, 10 μg/mL leupeptin and 10 μg/mL pepstatin A]. After the protein concentration was determined, each cell lysate was dissolved in sodium dodecyl sulfate (SDS)-Laemmli sample buffer [62.5 mM Tris–HCl (pH 6.8), 2% SDS and 10% glycerol]. Equal amounts of cell lysate were separated on 12.5 or 15% SDS–polyacrylamide electrophoresis gels, immunoblotted onto polyvinylidene difluoride membranes (Merck Millipore, Germany) and identified by an ECL Detection System (GE Healthcare Bioscience, U.S.A.) or Western Blotting Substrate Plus (Thermo Fisher Scientific, U.S.A.) using antibodies against Chac1 (1:1,000), c-Myc (1:1,000), HA (1:1,000), LC3 (1:3,000) or Actin (1:5,000). Band intensities were analyzed by Image J software (National Institutes of Health, U.S.A.).

### Immunoprecipitation

Transfected HEK293 cells in 60 mm dishes were harvested and lysed in lysis buffer [20 mM Tris-HCl (pH 8.0) containing 150 mM NaCl, 1% Nonidet P-40, 1 mM EDTA, 1 mM PMSF, 10 μg/mL leupeptin, and 10 μg/mL pepstatin A]. Lysates were cleared by centrifugation for 6 min at 12,000 × g and soluble proteins were immunoprecipitated using 2 μg of c-Myc antibody and Protein G Sepharose (GE Healthcare Bioscience, U.S.A.). Proteins binding to the resin were washed three times in wash buffer [20 mM Tris-HCl (pH 8.0) containing 150 mM NaCl, 0.2% Nonidet P-40, 1 mM EDTA and 1 mM PMSF], eluted by SDS-Laemmli sample buffer and subsequently analyzed by western blot.

### Measurement of glutathione

The glutathione content in cells was measured fluorometrically according to the method of Hisson and Hilf^57^. In brief, cells were cultured in Dulbecco’s Modified Eagle’s Medium and collected by centrifugation. Cell pellets were resuspended in 0.1 M sodium phosphate buffer (pH 8.0) containing 5 mM EDTA and 25% (w/v) metaphosphoric acid solution. After centrifugation for 10 min at 10,000 × g, each supernatant was incubated with 0.1 M phosphate buffer (pH 8.0) containing 5 mM EDTA and 0.1% *o*-phthalaldehyde for 15 min. Fluorescence of each sample was measured by fluorospectrometry using excitation at 350 nm and emission at 420 nm.

### Proteasome activity assay

The chymotryptic proteasomal activity including 26S proteasome activity in transfected HEK 293 cells was measured fluorometrically according to the method of Mark FP *et al.*^58^. In brief, the cells were cultured in Dulbecco’s Modified Eagle’s Medium and collected by centrifugation. The cell pellets were resuspended in lysis buffer [PBS containing 1% (v/v) Triton X-100] and incubated for 30 min on ice. After centrifugation for 15 min at 16,500 × g, each supernatant was incubated with assay solution [30 mM Tris-HCl (pH 7.6) containing 2 mM MgCl_2_, 10 mM NaCl, 10 mM KCl, 0.5 mM dithiothreitol, 1 mM ATP, and 100 μM Suc-LLVY-MCA] for 60 min at 37 °C in the presence or absence of 20 μM MG132. Fluorescence of each sample was measured by fluorospectrometry using excitation at 360 nm and emission at 450 nm.

### Statistical analysis

The results were expressed as the means ± SD of the indicated number. Statistical analysis was carried out by one way-ANOVA followed by Tukey’s test. **p < 0.01 was considered to be statistically significant.

## Additional Information

**How to cite this article**: Nomura, Y. *et al.* Translational and post-translational regulation of mouse cation transport regulator homolog 1. *Sci. Rep.* **6**, 28016; doi: 10.1038/srep28016 (2016).

## Supplementary Material

Supplementary Information
