# Supplementary material for: Translational and post-translational regulation of mouse cation transport regulator homolog 1
Source: Sci Rep. 2016 Jun 15;6:28016. doi: 10.1038/srep28016 (PMC4908420; doi:10.1038/srep28016)
Supplement: Supplementary Information [file srep28016-s1.pdf]

Supplementary Information for

Translational and post-translational regulation of mouse cation transport regulator homolog 1

Yuki Nomura[1], Yoko Hirata[1, 2], Kazutoshi Kiuchi[1, 2], Kentaro Oh-hashi[1, 2]*

[1]United Graduate School of Drug Discovery and Medical Information Sciences, Gifu University, 1-1 Yanagido, Gifu 501-1193, Japan.
[2]Department of Chemistry and Biomolecular Science, Faculty of Engineering, Gifu University, 1-1 Yanagido, Gifu 501-1193, Japan.

*Corresponding author: Kentaro Oh-hashi, Department of Chemistry and Biomolecular Science, Faculty of Engineering, Gifu University, 1-1 Yanagido, Gifu 501-1193, Japan.
Tel; +81-58-293-2659; Fax; +81-58-293-2794; E-mail: oohashi@gifu-u.ac.jp

Supplementary Materials and Methods

RT-PCR.
To estimate the expression level of each gene by RT-PCR, total RNA was extracted from the transfected cells lysed with TRIzol and treated with DNase. After converting the RNA to cDNA by reverse transcription using random ninemers to prime SuperScript III Reverse Transcriptase (RT) (Life Technologies, U.S.A.) as previously described[28]. Each cDNA was added to a PCR reaction mixture for amplification (Taq PCR kit, Takara, Japan). For evaluation of extrinsic Chac1 mRNA, T7 primer and Chac1 antisense primer (5'-CTGTGTGGCAATGACCTCTTC-3') were used. Expression of glyceraldehyde 3-phosphate dehydrogenase (G3PDH) mRNA was detected using following primer pairs: sense primer 5'-ACCACAGTCCATGCCATCAC-3'; and G3PDH antisense primer 5'-TCCACCACCCTGTTGCTGTA-3'. The typical reaction cycling conditions were 30 sec at 96℃, 30 sec at 60℃ and 30 sec at 72 ℃. The results represent 20-34 cycles of amplification, and the resulting products were separated by electrophoresis on 2.0% agarose gels and visualized using ethidium bromide. The experiments were repeated to confirm reproducibility. The expression level of extrinsic Chac1 gene was analyzed by Image J software (National Institutes of Health, U.S.A.) and normalized by values obtained from cells expressing Chac1-Myc lacking its 5'UTR.

```
mouse  -------------------------GGCCTTCCTGGCCGGAGGGTTTAAAGAGCGCCTCTGGAGG
rat    CCGGGACAGCTGGAGGCAGTCAGAGGGCCTTCCTGGCCGGAGGGTTTAAAGAGCGCCTCTGGAGG
human  ----------------------------------------AAGGTTTAAAGGGCGCCTCCATGGG
                                               * ********* *******    **

       GACCACTCAGCTGGAACGACCGATCGGTGCCAGGCCAGGTGTACGCGTCCGTCGGTCCTTCCGTG
       GACCACTCAGCTGGAACGACCGATCGGTGCCAGGCCAGGTGTACGCGTCCGTCGGTCCTTCCGTG
       GGGCGCTCAGCTGGAGCTACCGAGCGGTGCCAGGCCAGGTGTGTGCGTCCGTCGGTCTTTCCGTG
       *  * ********** * ***** ******************  ************* *******

                                                        Kozak-like
       CCCGTGCCGGAGACCAGTCTCGGAGGCCACCCGGGTCCGTCCCTGCGCCCGGCACCATG
       CCCGTGCCGGAGACCAGCCCCGGAGGCCGCCCGGGCCCGTCCCTGCGCCCGGCACCATG
       CCCACGCCGGAGACCAGCCCCGGAGGCCGCCTGGGCCTATCCCTGTGCCAGGCACCATG
       ***  ************ * ******** ** *** *  ****** *** *********
```

Supplementary Figure S1. Nucleotide sequences of 5’UTR of mouse, rat and human Chac1 genes.

The nucleotide sequences were referred to the NCBI Reference Sequences, NM_026929, NM_001173437 and NM_024111, respectively . Nucleotide sequences conserved between the 5’-flanking promoter region of the mouse, rat and human Chac1 genes are indicated with asterisks. The conserved translational start codon and the ATG only in the human Chac1 5’UTR are highlighted in red and blue, respectively. The upstream ATG in the human Chac1 gene is suggested to be unfunctional. The conserved Kozak-like sequence just before the translational start site is shown in a box.

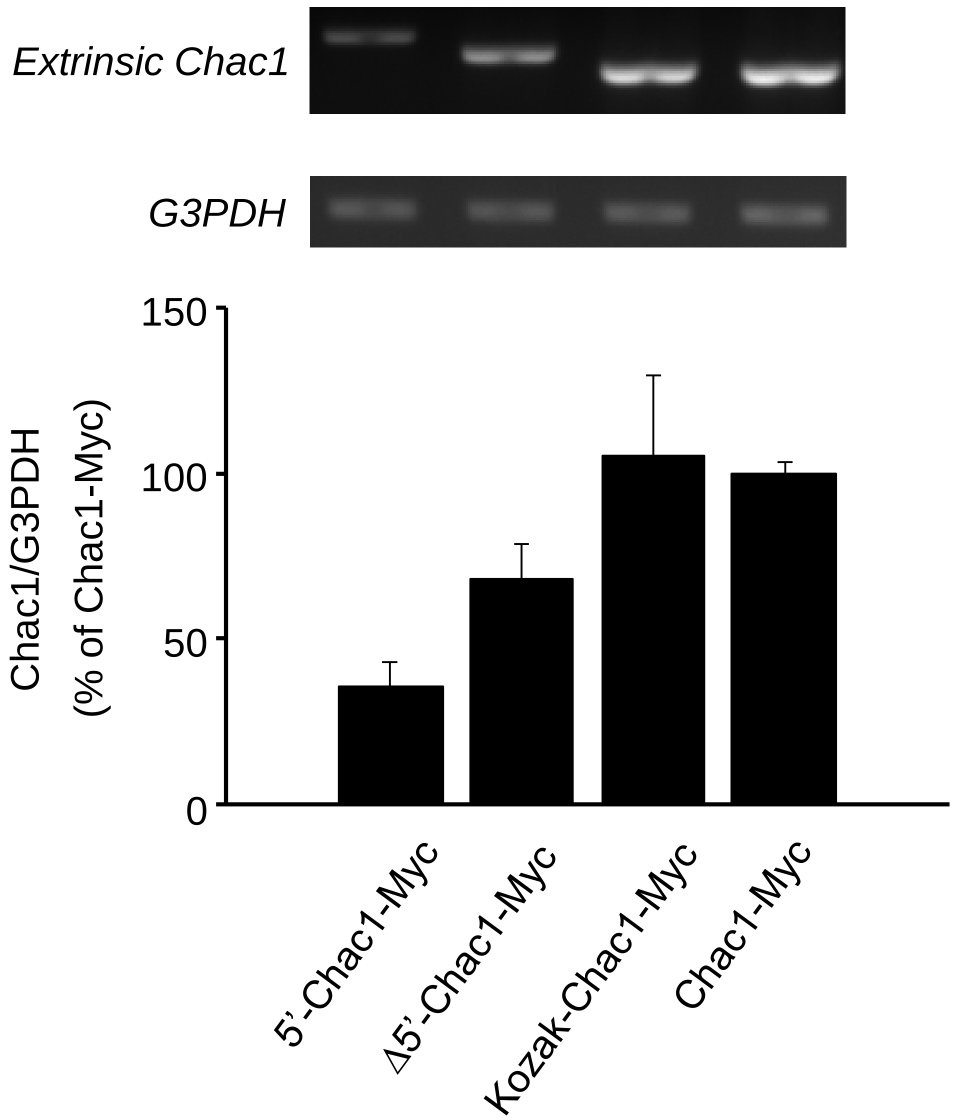

Supplementary Figure S2. The expression of Chac1 mRNA derived from each of Chac1 constructs in HEK293 cells.
Thirty-six hours after transfection with the indicated genes, its expression was determined by RT-PCR as described in the Supplementary Materials and Methods. Relative amounts of extrinsic Chac1 mRNA were calculated and values represent the mean ± SD obtained from three independent cultures.

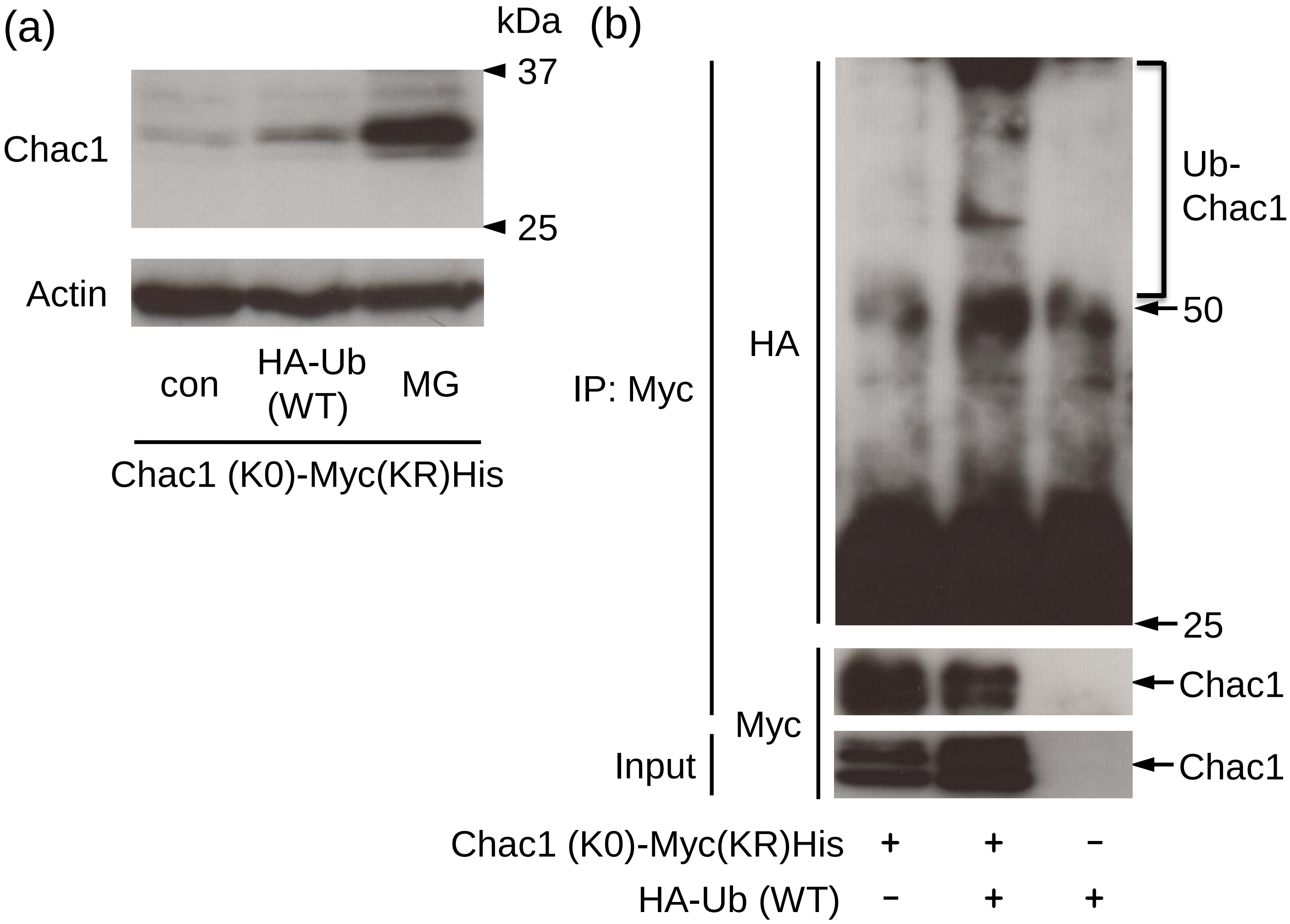

Supplementary Figure S3. Chac1 (K0)-Myc(KR)His is up-regulated by HA-Ub (WT) overexpression and actually ubiquitinated.
(a) HEK293 cells were transiently co-transfected with Chac1 (K0)-Myc(KR)His and HA-Ub (WT). After 24 h, the cells were treated with vehicle (con) or 20 μM MG for 12 h, and the resultant cell lysates were analyzed by western blot using c-Myc or Actin antibodies. (b) HEK293 cells were transiently co-transfected with Chac1 (K0)-Myc(KR)His and HA-Ub (WT). After 24 h, the cells were treated with 20 μM MG for 12 h. Myc(KR)-tagged proteins were immunoprecipitated using c-Myc antibody and analyzed by western blot using HA or c-Myc antibodies. The total cell lysate (Input) was analyzed by western blot using c-Myc antibody. Ub-Chac1 indicates the ubiquitinated Chac1.
